# Applications of CRISPR/Cas9 in the research of malignant musculoskeletal tumors

**DOI:** 10.1186/s12891-021-04020-2

**Published:** 2021-02-05

**Authors:** Wei Liu, Shubin Wang, Binhui Lin, Wei Zhang, Guangrong Ji

**Affiliations:** 1 Department of Orthopaedics, Xiang’an Hospital, School of Medicine, Xiamen University, No. 2000 East Xiang’an Road, Xiang’an District, Xiamen, 361102 China; 2 Department of Obstetrics and Gynecology, Union Hospital, Tongji Medical College, Huazhong University of Science and Technology, Wuhan, 430022 China

**Keywords:** CRISPR/Cas9, Gene editing, Osteosarcoma, Ewing’s sarcoma, Rhabdomyosarcoma

## Abstract

**Background:**

Malignant tumors of the musculoskeletal system, especially osteosarcoma, Ewing sarcoma and rhabdomyosarcoma, pose a major threat to the lives and health of adolescents and children. Current treatments for musculoskeletal tumors mainly include surgery, chemotherapy, and radiotherapy. The problems of chemotherapy resistance, poor long-term outcome of radiotherapy, and the inherent toxicity and side effects of chemical drugs make it extremely urgent to seek new treatment strategies.

**Main text:**

As a potent gene editing tool, the rapid development of CRISPR/Cas9 technology in recent years has prompted scientists to apply it to the study of musculoskeletal tumors. This review summarizes the application of CRISPR/Cas9 technology for the treatment of malignant musculoskeletal tumors, focusing on its essential role in the field of basic research.

**Conclusion:**

CRISPR, has demonstrated strong efficacy in targeting tumor-related genes, and its future application in the clinical treatment of musculoskeletal tumors is promising.

## Background

Clustered regularly spaced short palindromic repeats, i.e.,The CRISPR sequence was initially identified in *E. coli* [1]. and was later confirmed to be widespread in bacteria and archaea. The sequence consists of nonadjacent identical sequences (repeats) and variable sequences (spacers) that are similar in size. The CRISPR sequence is adjacent to the CRISPR related gene (CAS9) and together constitutes the CRISPR/CAS9 system, CRISPR, is an important component of the immune defence system of prokaryotes, providing them with adaptive immunity against phage infection and plasmid transfer [2–4]. The CRISPR sequence can produce mature CRISPR RNA (crRNA) after transcriptional and enzymatic processing [5]. The repeated sequence of crRNA with CAS nuclease pairs with trans-activated crRNA (tracrRNA) bases to form a complex with a double-RNA hybrid structure. The crRNA spacer can be integrated with the exogenous phage genome spacer (prospacer) to enable the bacteria to form an immune memory of the exogenous gene, thereby increasing the host’s phage resistance [6]. CAS9 can then be directed to specific target DNA loci for cleavage to produce a blunt-ended double-strand breaks (DSBs) [7], In mammalian cells, DSBs are then repaired by either homology-directed repair (HDR) [8] or nonhomologous end joining (NHEJ) [9]. Due to its high fidelity, HDR can perform precise genetic repair, whereas NHEJ performs error-prone and inaccurate repairs by generating random deletions or insertions at the break site.

**Fig. 1:**
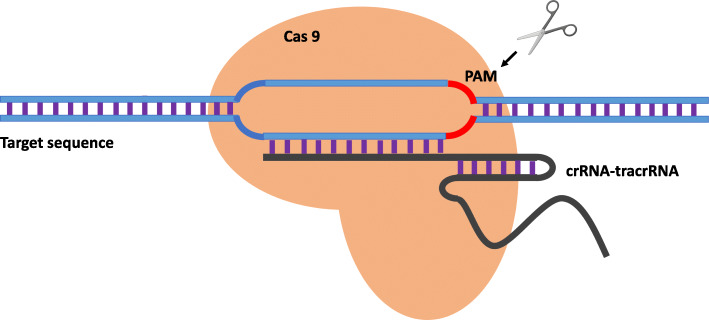
The basic working mechanism of CRISPR/CAS9 in mammalian cells. The crRNA-tracrRNA fusion transcript (gRNA) combines with the CAS9 protein to form a complex that targets the DNA sequence and knocks out target genes

Beyond an important role in prokaryotes, CRISPR/CAS9 gene editing technology was applied for mammalian cell genome editing by Mali [10] and Cong [11]. et al. for the first time in 2013, by modifying the bacterial CRISPR system to allow mammalian cells to heterologously express key components of the prokaryote CRISPR system or an artificially designed crRNA-tracrRNA fusion transcript (gRNA),which can direct CAS9 to the target DNA sequence and perform targeted cleavage to achieve gene knockout. The basic working mechanism of CRISPR/CAS9 in mammalian cells is shown in Fig. 1. Since then, CRISPR/CAS9 technology has quickly become a research hotspot in the basic sciences and biomedical fields. Generally, CRISPR/CAS9 technology is superior to ZFN and TALEN gene editing techniques widely used in the past based on the following aspects: 1. The design is simpler. With the same CAS9 nuclease, the new DNA target sequence can be edited again only by replacing the sgRNA sequence, which saves time [12, 13]. 2. Higher gene editing efficiency. sgRNAs are relatively short and multiple sgRNAs can be introduced to achieve simultaneous editing of multiple target genes [14]. 3. Higher targeted binding efficiency and lower cost [13]. These unique advantages of CRISPR/CAS9 technology have promoted its use in research in the field of biomedicine and have made major breakthroughs in the clinical treatment of human diseases. For example, scientists engrafted *CCR5-*knockout HSPCs by CRISPR/CAS9 technology into patients with both acute lymphocytic leukemia (ALL) and HIV infection. Complete remission of ALL was achieved without adverse events related to gene editing, and the percentage of CD4+ cells ablated by *CCR5* in this patient was increased after discontinuing antiretroviral treatment [15]. In addition, CRISPR/Cas9 technology has now been widely applied in various clinical trials for treating human diseases such as blood diseases, hereditary eye diseases, viral diseases and cancers, including malignant glioma, metastatic non-small cell lung cancer, prostate cancer, oesophageal cancer, and renal cell cancer [16]..

Malignant musculoskeletal tumors most commonly occur in adolescents and children, and osteosarcoma, Ewing’s sarcoma (ES) and rhabdomyosarcoma exhibit the highest incidences. The current main treatment methods include surgery, chemotherapy and radiation therapy [17]. The application of combination therapy has improved the prognosis of patients compared with previous single surgical resection, but the toxicity and side effects of chemotherapy drugs, the risk of long-term tumor recurrence and new cancers induced by radiotherapy, mean that new treatments with low toxicity side effects and better long-term efficacy are urgently needed. The benefits of CRISPR/CAS9 technology in basic research and clinical applications in various human diseases have promoted its exploration in musculoskeletal tumors, including screening and knockout of oncogenes or drug-resistant genes, identification of tumor suppressor genes and construction of cell or animal tumor models. An in-depth understanding of the application status and application challenges of CRISPR/CAS9 technology in the study of malignant musculoskeletal tumors will help accelerate the research progress of these tumors. In this review we describe the recent exciting progress in the use of CRISPR/Cas9 technology in the race to develop treatments for malignant musculoskeletal tumors.

### Osteosarcoma and CRISPR/CAS9

**Fig. 2:**
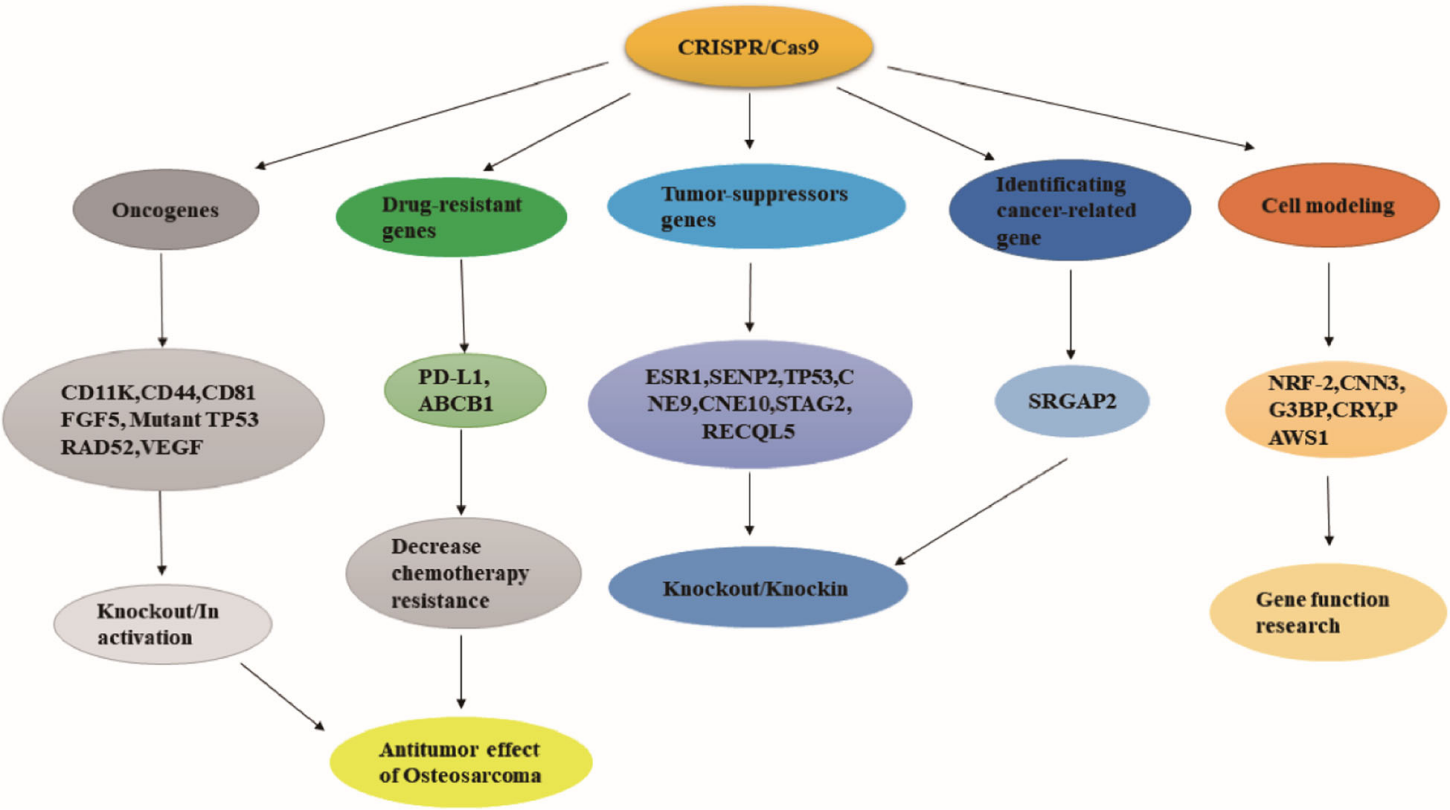
The application of CRISPR/Cas9 in osteosarcoma research. The applications of CRISPR/Cas9 in the research of osteosarcoma primarily involve in oncogene knockout, drug resistance gene knockout, tumor suppressor gene knockout or knock-in, cancer-related gene identification and cell modelling

Among skeletal tumors in adolescents and children, osteosarcoma has the highest incidence, ranking third among all childhood tumors, second only to lymphoma and brain cancer, and accounting for approximately 1% of adult tumors [18, 19]. Only 800 new cases are reported annually in the U. S, and the prognosis for patients with osteosarcoma is often unsatisfactory despite the relatively low incidence [18]. The overall ten-year survival rate is only approximately 50% [20, 21]. It is worth noting that 30 to 40% of patients with local tumors will relapse after treatment [19], greater than 80% of the local tumor may metastasize, and approximately 20% of tumors had metastasized when the disease is diagnosed [18]. For those with metastasis or relapse, less than 30% survived in the next five years [19]. The current treatment plan is mainly neoadjuvant chemotherapy plus surgery combined with postoperative chemotherapy for at least 6 to 8 months. The preferred chemotherapy regimen is a MAP combination regimen consisting of cisplatin, high-dose methotrexate, and Adriamycin [18, 21, 22]. The use of combined chemotherapy has improved patient survival to some extent, but the toxic side effects of chemotherapy drugs have aroused people’s concerns, such as the cardiotoxicity of doxorubicin [23], the otorenal toxicity and carcinogenicity of cisplatin [24–26], methotrexate-induced bone marrow suppression and liver, kidney, and ocular mucosal toxicity [25, 26].. Another concern is the resistance of tumors to chemotherapy, which often causes disease recurrence and reduces patient survival [27]. To overcome these clinical problems, we need to understand the pathogenesis of osteosarcoma and control it at the genetic level. Although the exact aetiology of osteosarcoma is unknown, there is evidence that hereditary diseases such as hereditary retinoblastoma, Li-Fraumeni syndrome, Rothmund-Thomson syndrome and Werner syndrome may affect the occurrence of osteosarcoma, and the disease-causing genes of these syndromes are *RB1*, *TP53, RECQL4* and *WRN*, suggesting that abnormalities in these genes may form part of the pathogenesis of osteosarcoma [18, 28]. In addition, germline genes and pharmacogenetics also profoundly affect the treatment of osteosarcoma, including genes related to drug transport and DNA repair, such as *ABCC3*, *ABCB1*, *RFC1*, *GST* family, *ERCC1*, *ERCC2* and *XPC*. *ABCC3*, *ABCB1*, *RFC1*, and *GST* are genes encoding drug transporters, whereas *ERCC1*, *ERCC2*, and *XPC* are involved in the repair of DNA damage induced by cisplatin. Many studies have demonstrated that mutations in these genes affect drug sensitivity in patients with osteosarcoma and are often associated with poor prognosis [29–36]. Because CRISPR/CAS9 can precisely target pathogenic genes, its application in the treatment of osteosarcoma is promising. In recent years, some progress has been made in CRISPR/CAS9 technology in the area of osteosarcoma (summarized in Table 1), and its major applications are presented in Fig. 2.

**Table 1:** The application of CRISPR/Cas9 in osteosarcoma research

Target Genes	Cell lines	CRISPR/Cas9 Applications	Effects	References
*CD11K*	KHOS,U2OS	knock out	Proliferation↓, Migration↓, Invasion↓, cell death↑	[37]
*GLT25D1*, *GLT25D2*	Saos2	knock out	Non-survival cells	[38]
*CD44*	MNNG/HOS, 143B	knock out	Migration↓, Invasion↓, spheroids formation↓	[39]
*CD81*	143B	knock out	Tumors growth in mice↓ lung metastases↓	[40]
*FGF5*	MG63,U20S	knock out	Proliferation↓, tumor growth in mice↓, MAPK pathway activity↓	[41]
mutant *TP53*	KHOS,KHOSR2	knock out	Proliferation↓,migration↓, clony formation↓, IGF1-R↓,Bcl2↓,Survivin↓, doxorubicin sensitivity↑	[42]
*PD-L1*	MNNG/HOS, KHOS	knock out	Chemoresistance to doxorubicin and paclitaxel↓	[43]
*CD44*	KHOSR2, U-2OSR2	knock out	Migration↓,Invasion↓, spheroids formation↓, doxorubicin sensitivity↑	[44]
*ABCB1*	KHOSR2, U-2OSR	knock out	doxorubicin sensitivity↑	[45]
*CNE9,* *CNE10*	U2OS	knock out	Proliferation↑,apoptosis↓, SHOX expression↓	[46]
*STAG2*	U2OS	knock out	Proliferation↓,EMT↑,migration,cisplatin chemoresistance↑,PDL1,CDK4,RB expression↑,CCNB1,CCND1,CDK1↓,G2/M arrest, PI3k/AKT Pathwayactivity↓	[47]
*ESR1*	143B	knock out	Proliferation↑,osteoblast differentiation↓,tumor growth and metastasis in mouse↑ VIMENTIN,SLUG,ZEB1,MMP9,SOX2,OCT4,NANOG expression↑	[48]
*SENP2*	HOS	knock out	Proliferation↑,migration↑,invasion↑,SOX9 expression↑	[49]
*RECQL5*	MG-63	knockin	Proliferation↓,apoptosis↑,cell cycle arrest,bcl-2↓,caspase-3↑	[50]
*TP53*	porcine zygotes	knock out	Genetical porcine model with mandibular osteosarcoma	[51]
*RAD52*	U2OS	knock out	Tumor Growth↓,lifespan↑, DNA replication↓	[52]
*PAWS1*, *CD2AP*	U2OS	knock out knockin	Migration↓,Focal adhesions↓,cell adhesion ability↓	[53]
*NRF2*	U2OS	knock out	No function research	[54]
*Cnn3*	U2OS	knock out	Stress fiber networks organizations and contractility abnormalities. stress fiber breakage events↑	[55]
*G3BP*	U2OS	knock out	For studying potential proviral roles of G3BP	[56]
*CRY1,CRY2*	U2OS	knock out	For studying circadian rhythms in mouse	[57]
*SRGAP2*	K12	knock out	Migration↑	[58]
*VEGF*	K7M2	knock out	Proliferation↓, Migration↓, Invasion↓ in vivo and vitro.	[59]

Notes: ↑:Up-regulation: ↓:Down-regulation

Feng. et al. first applied CRISPR/CAS9 technology to osteosarcoma research in 2014 when they knocked out the CD11K genes of KHOS and U-2OS human osteosarcoma cells, and demonstrated significant inhibition of migration, invasion activity and cell proliferation [37]. Since then, the CRISPR/CAS9 technique has been widely applied for osteosarcoma oncogene knockout. A study showed that human Saos osteosarcoma cells were nonviable when the *GLT25D1* and *GLT25D2* genes were knocked-out [38]. *CD44* knockout suppressed the mobility of 143B and MNNG/HOS cells, both of which are highly invasive human osteosarcoma cell lines, and their spheroid formation and growth were also significantly inhibited [39]. In addition, CRISPR/CAS9 gene-edited cells also exhibited similar effects in vivo. In one study, *CD81* knock-out 143B cells were injected into nude mice, and the occurrence of pulmonary metastases was significantly reduced compared with the control group [40]. Another study used MG63 and U2OS cells in which the FGF5 gene was knocked out to perform tibia in situ transplantation in nude mice, and tumor growth in vivo was significantly inhibited [41]. It is worth noting that CRISPR/CAS9 technology can be applied to improve the chemosensitivity of osteosarcoma cells. KHOSR2, a multidrug-resistant osteosarcoma strain, exhibited increased sentivity to Adriamycin by mutant *TP53* knockout [42]. *PD-L1-*knockout made KHOS and MNNG/HOS cells more sensitive to paclitaxel and doxorubicin [43]. For KHOSR2 and U-2OSR2, the expression of the drug efflux protein p-gp encoded by ABCB1 was significantly reduced with increased doxorubicin absorption and sensitivity after CD44-knockout by CRISPR/CAS9 technology [44]. When direct knockout of the *ABCB1* gene was performed in KHOSR2 and U-2OS cells, the chemoresistance of these cells to Adriamycin was effectively reversed [45]. These experiments suggest that targeted oncogene knockout via CRISPR/CAS9 technology has the potential to inhibit osteosarcoma progression, and hopefully overcome the problem of chemoresistance.

In addition to knocking out oncogenes, CRISPR/CAS9 technology was also used to edit cancer suppressor genes to verify their biological roles. The CRISPR/CAS9-mediated knockout of *CNE9, CNE10* or *STAG2* gene could decrease U2OS cell apoptosis. *CNE9* or *CNE10* knock-out U2OS cells exhibited significantly inhibited cell proliferation, suggesting the cancer suppressor roles of both genes, For *STAG2-*knockout cells, the epithelial-mesenchymal transition (EMT), cell invasion and PD-L1 gene expression were obviously promoted, whereas expression changes of many immune-related genes and increased chemo-resistance to cisplatin were also observed, indicating that the absence of *STAG2* may protect tumor cells from attack by the immune system mediated by PD-L1, which may offer evidence that *STAG2* is possibly a new potential biomarker for *PD-1*-PD-L1 inhibitor therapy in *STAG2*-deficient osteosarcoma [46, 47]. Decitabine (DAC), a DNA methylation inhibitor can inhibit in vitro cell proliferation, mobility, anchoring independence, and spheroid formation, reduce in vivo xenograft tumor growth and metastasis and reduce the expression of tumor stem cell markers such as *SOX2, OCT4, NANOG*, and CRISPR/CAS9-mediated *ESR1* gene knockout, effectively eliminating the abovementioned effects of DAC, and demonstrating that the inhibitory effect of DAC on osteosarcoma depends on the presence of the *ESR1* gene [48]. Using the CRISPR/Cas9 technique, scientists also demonstrated that SENP2, which was expressed at lower levels in primary human osteosarcoma tissue and cell lines, may be a potential target for osteosarcoma treatment. In their study, engineered SENP2 overexpression significantly inhibited cell proliferation, migration and invasion, which was reversed by CRISPR/Cas9-mediated *SENP2*-knockout [49]. Similar to gene knockout, CRISPR/Cas9 technology was also applied to construct stable gene overexpression cell lines. *RECQL5*, a gene that is downregulated in osteosarcoma tissue and cells, was inserted into human AAVS1 safe harbor using the CRISPR/Cas9 system to investigate its function in osteosarcoma progression. A stable RECQL5 overexpression MG63 cell line was constructed, and significant cell proliferation inhibition, cell cycle arrest and apoptosis promotion were observed, suggesting *RECQL5* that is a tumor suppressor in osteosarcoma [50].

CRISPR/CAS9 was also used to construct animal or cell models of human osteosarcoma. Tanihara. et al. performed targeted *TP53* knockout of pig in vitro zygotes, and half of the live piglets produced after in vivo transplantation developed various tissue tumors, including osteosarcoma. The combination of CRISPR/Cas9 technology and in vitro fertilization technology can effectively reduce the research cost of generating gene-mutant pigs, and generate a pig osteosarcoma model that is similar to the physiological anatomy and genetics of humans, which will certainly provide great help for preclinical osteosarcoma research [51]. In addition, the construction of a *RAD52*-knockout U2OS cell model through CRISPR/Cas9 technology helped to demonstrate that *RAD52* is an important gene for repairing the collapsed DNA replication forks damaged by oncogenes or chemicals in cancer cells, given that *Rad52* knockout by CRISPR/Cas9 compromised the restarting of collapsed forks and led to DNA damage [52]. The construction of *PAWS1*- and *CD2AP*-knockout U2OS cell models demonstrated that *PAWS1* and *CD2AP* play essential roles in cell actin organization and focal adhesion, thus affecting cell adhesion and migration. Specifically, *PAWS1* or *CD2AP* knockout by CRISPR/CAS9 resulted in disorganized and tangled actin mesh, reduced migration ability, and failure to properly form focal adhesions. These findings are beneficial to broaden the research onbiochemical and molecular mechanisms of cytoskeleton structure regulation, and are of great significance for deeply understanding the process of embryonic development, angiogenesis, epithelial-mesenchymal transition and cancer migration [53]. Moreover, some other cell models, such as *NRF-2-*knockout U2OS for determining the inhibitory mechanism of sulforaphane on mTOR [54].,*CNN3*-knockout U2OS for investigating its role in stress fibre assembly and contractility [55],*G3BP*-knockout U2OS for studying potential pro-viral roles of G3BP [56], and *CRY1,CRY2*-knockout U2OS cell models for studying human circadian clocks [57].

The function of CRISPR/CAS9 technology in screening and identifying cancer-related genes deserves attention and further development. For example, Marko. et al. used the cancer gene database to perform forward genetic screening of cancer-driving genes, and screened for the genetic locus *SRGAP2* that may be related to the malignant progression of osteosarcoma. Then, they used CRISPR/Cas9 and doxycycline to knock out and overexpress *SRGAP2* respectively in murine osteosarcoma cell lines, and found that *SRGAP2* knockout increased cell migration, whereas *SRGAP2* overexpression reduced cell migration, demonstrating that *SRGAP2* may act as a migration inhibitor [58]. Some researchers performed candidate gene analysis, pathway-based gene analysis and genome-wide association studies (GWAS) to screen out pharmacokinetics-associated genes of MAP therapy (i.e., drug metabolism enzymes genes GSTT1,GSTM1, GSTM3*B, and *GSTP1,* and transporters genes *ABCB1,ABCB3,*and *RFC1*) and pharmacodynamic genes associated with cisplatin treatment(i.e.,DNA damage repair genes *REV1,REV3,ERCC1,ERCC2,ERCC5,*and *XPC*) [26]. If CRISPR/CAS9 technology was used for corresponding gene editing, it might have provided more promising molecular targets for osteosarcoma treatment, meanwhile. By identifying more genes or signalling pathways that affect drug efficacy, we can further study whether and how such genes are involved in the genesis and progression of osteosarcoma.

In short, osteosarcoma has a high risk of metastasis and is prone to chemotherapy resistance, and the outcomes of treatment for patients have not improved in decades. The extensive application of CRISPR/CAS9 may solve the above problems in the near future.

### Ewing’s sarcoma and CRISPR/CAS9

Ewing’s sarcoma is also a common malignancy of the skeletal system, second only to osteosarcoma in incidence, with a rate of 2.93/1 million in the United States for a period of past 30 years. Between 2000 and 2005, an average of 208 new patients were added annually, and greater than one quarter had metastasized at diagnosis [60]. The 10-year survival rate for non-metastatic patients is approximately 66.8%, compared with 28.1% for metastatic patients [61]. Unlike osteosarcoma, ES has a clear genetic aetiology. Specifically, the *EWSR* gene of chromosome 22 and the ETS family gene of chromosome 11 are translocated to form a fusion gene, and the ETS family gene includes *FL1,ERG,ETV1,ETV4,* and *FEV.* In most cases, *EWSR* is fused with the *FL1* gene, accounting for approximately 85% of cases. The ETS family genes are responsible for encoding transcription factors, enabling fusion genes to have powerful transcriptional activation activities to induce the genesis of ES [62, 63]. In addition, many other genes were also reported to promote the occurrence and progression of ES, such as the human telomerase reverse transcriptase gene, *VEGF*, *IGF-1*, *CAV1*, *EZH2*, *BMI1*, *NKX2.2*, *NROB1*, *GLI1*, *RB* and *p53* [62] .

Current treatments for ES include surgery, radiotherapy and standard chemotherapy consisting of vincristine, doxorubicin, cyclophosphamide, ifosphamide, and etoposide [63]. As the EWSR1-FLI1 fusion protein is located in the nucleus and is difficult to attack directly, traditional chemotherapeutics targeting EWSR1-FLI1 often fail to achieve satisfactory results [64, 65]. Although multimodal treatment strategies have improved patient survival rates to some extent, many problems still exist. These problems are mainly reflected in the low long-term survival rate of survivors, tumor metastasis or recurrence, chemoresistance, and the relatively increased risk of secondary malignancies [62, 66, 67]. According to a national study of ES survivors with greater than 30 years of follow-up in Sweden, the risk of breast cancer is significantly increased by approximately 4.7 times that in the general population, whereas the risk of secondary soft tissue sarcoma is 67 times that in the general population [68]. Cohort studies of 23,603 5-year survivors with childhood tumors in the U. S and Canada show that radiotherapy is an important factor in secondary malignancies [66]. These studies indicate that the long-term outcome of ES patients is not ideal, thus new treatment methods are urgently needed. In recent years, scientists have begun to apply CRISPR/Cas9 technology to ES research, and some progress has been made (summarized in Table 2).

**Table 2:** The application of CRISPR/Cas9 in Ewing’s sarcoma research

Target Genes	Cell lines	CRISPR/Cas9 Applications	Effects	References
*EWSR1*,*FLI1*	hMSC,hiPSC,HEK293A,HEK293T	induce chromosomal translocation	EWSR1-FLI1 chromosomal translocation cell model	[69–71]
*MDM2*,*MDM4*,*TP53*,*USP7*,*PPM1D*	TC32,TC138 A673,EWS502	knock out	Proliferation↓, chemical cytotoxity↓	[72]
*PHF19*	SK-N-MC	knock out	Proliferation↓,colony formation↓, invasion↓, JQ1 sensitivity↑	[73]
*TNC*	A673,SKNMC	knock out	Proliferation↓,migration↓, metastases in mice↓, Hippo/YAP pathwayactivity↓,MALAT1, P-SRC,P-MYC expression↓	[74]
*MSH2*	A673	knock out	microsatellite instability (MSI)↑, somatic mutation frequencies ↑, compound-resistant to cellular toxins: PSMB5,CD437,MLN4924↑	[75]

Notes: ↑:Up-regulation: ↓:Down-regulation

In 2014, CRISPR/Cas9 technology was first used to construct an ES cell model of EWSR1-FLI1 translocation mutations in HEK293 and human adult mesenchymal stem cells (hMSCs) - one of the origin cells of human ES, and EWSR1-FLI1 fusion protein expression was observed. Six genes targeted by the *EWSR1-FLI1* gene were also upregulated, demonstrating the potential of CRISPR/Cas9 technology for cancer modelling of ES [69]. To optimize the efficiency of CRISPR/Cas9, Torres-Ruiz. et al. developed the ssODN-RNP CRISPR/Cas9 coupling method to more efficiently generate t (11, 22) translocations in hMSCs and hiPSCs, promoting ES modelling via a combination of stem cell technology and CRISPR/CAS9 technology [70]. In the same year, Spraggon. et al. developed a novel method that combines CRISPR/Cas9 with HDR to engineer and modulate the expression of chromosomal translocation products, and allowed the expression of the *EWSR1-FLI1* fusion gene to be controlled in a timely manner, which effectively solved the problem that the permanent generation of the EWSR1-FLI1 fusion gene caused the expression change of its intracellular target gene in a short time and made them difficult to precisely target, This strategy is undoubtedly more conducive to the study of the genetic and pathogenic mechanisms of ES [71].

In the study of ES gene function, CRISPR/CAS9 technology also demonstrates great value. Stolte. et al. knocked out multiple genes including *MDM2, MDM4, PPM1D* and *USP7* in mutated TP53 and wild-type TP53 ES cell lines, and they found that cell viability was significantly reduced only in wild-type TP53 cells, suggesting that the presence of wild-type TP53 may be a prerequisite for these oncogenes to play carcinogenic roles. In addition, CRISPR/Cas9 technology was applied for screening cancer-related genes and exploring effective gene-targeted drug combinations in this study. For example, they used CRISPR/Cas9 to edit a number of genes and eventually found that the *MDM2/MDM4* double knockout exhibited an increased antitumor effect. Then they combined the *MDM2/MDM4* dual inhibitor, ATSP-7041 with doxorubicin, etoposide and vincristine, and achieved a more significant growth inhibition of tumor in xenograft mice than control mice, which provides a theoretical basis for the combination of ATSP-7041 and conventional chemotherapy drugs in clinical use for ES patients [72]. Another study showed that *PHF19*-knockout significantly reduced cell proliferation, colony formation and invasion capacity, and increased the sensitivity of SK-N-MC ES cells to the BET bromodomain protein inhibitor JQ1 which can reduce proliferation and induce apoptosis of ES cells. This study also revealed the potential of JQ-1 for clinical utility in treating ES [73]. Similarly, *TNC*-knockout using the CRISPR/Cas9 system in A673 and SKNMC cells significantly inhibits cell proliferation, migration and angiogenesis. After injecting *TNC*-knockout cells into nude mice, their ability to transfer and colonize in vivo was significantly reduced compared with that of the control [74]. A recent study produced a defective DNA mismatch repair (dMMR) phenotype by knocking out the *MSH2* gene in A673 cells, which increased the cell’s gene mutation rate, and was used as a forward genetics system to uncover compound targets. Specifically, compound-resistant mutant clones can be obtained after treatment with three cellular toxins, namely, PSMB5, CD437 and MLN4924. This approach which promotes the appearance of compound resistance alleles through CRISPR/Cas9-mediated dMMR can be applied to identify the mechanism of anticancer effects of compounds screened out by phenotypic small molecules, and to model the genetic mechanism of chemoresistance in currently used anticancer therapies [75].

Currently, the use of CRISPR/CAS technology for ES research has only just begun. Although many cell models have been established, effective animal models remain lacking. Therefore, further advancement in animal modelling and cancer-related genetic modification (such as EWS/ETS target genes) of ES by CRISPR/Cas9 technology is needed, and it is expected to provide more options for clinical treatment of ES.

### Rhabdomyosarcoma and CRISPR/CAS9

Among human soft tissue sarcomas, rhabdomyosarcoma has the highest incidence, accounting for approximately 4–5% of malignancies in children, histologically, it is mainly divided into embryonal sarcomas (ERSMs) and alveolar sarcomas (ARSMs), with the former accounting for approximately 80% and the latter accounting for approximately 15–20% [76]. Current treatments for RSM include chemotherapy, surgery, and radiation. Although multidisciplinary treatment strategies cure approximately two-thirds of nonmetastatic RSM patients, the poor prognosis of metastatic and recurrent RSM patients urgently needs to be improved, as their 3-year overall survival rate is only 34 to 56% [77, 78]. RSM is mainly derived from skeletal myoblasts. Genetically, ERMS is characterized by allelic deletions on chromosome 11, and the main genetic characteristics of ARSM are chromosomal translocation of 1, 13, and 2, 13, with the production of fusion genes *PAX7-FOXO1* or *PAX3-FOXO1*, respectively, which dysregulate various genes that participate in cell transcription and differentiation, thus promoting carcinogenesis. Therefore, ARSM is often more disruptive than ERSM [17, 79, 80]. In addition to fusion genes, many other genes were also considered to be involved in the development of RSM, such as *NRAS, KRAS, HRAS, PIK3CA, FBXW7, BCOR, FGFR4* and *CTNNB1* [81]. The precise gene modification function of CRISPR/CAS9 technology has enabled its application in RSM research. Current progress achieved by CRISPR/CAS9 technology in the construction of RSM cell models, cancer gene screening and gene function research. is summarized in Table 3.

**Table 3:** The application of CRISPR/Cas9 in Rhabdomyosarcoma research

Target Genes	Cell lines	CRISPR/Cas9 Applications	Effects	References
*Pax3,Foxo1*	Foxo1-inv+/+ myoblasts (mice)/primary myoblasts	induce chromosomal translocation	Pax3-Foxo1 chromosomal translocation ARSM model, no function research	[82]
*DMD*	CCL-136 RD	knock out	Produce DMD deletion - immortalized muscle cell line	[83, 84]
*DYSF*	TE671	knock out	Myogenin↓,TSP-1 expression↑,membrane Repair ability ↓,	[85]
Dozens genes	JR1,RD	knock out	Validates oncogenes and tumors suppressors defined by iExCN tool	[79]
*HDAC1–10* genes (class I and II HDAC genes)	381 T, RD,SMS-CTR, Rh3,Rh5,Rh30	knock out	cell growth↓, myogenic differentiation↑, xenografts tumor proliferation↓,differentiation↑,	[86]
*PAX3-FOXO1*	Dbt-MYCN / indP3F parental cells^a^, recurrent tumour-derived cells.	knock out	Fail to form tumor in Dbt-MYCN/indP3F parental cells, form tumor in recurrent tumour-derived cells	[80]
*NRAS,HRAS*	381 T,SMS-CTR ERMS cells	knock out	Cell viability↓, apoptosis↑, G1 phase arrest, expression of key cell-cycle and DNA replication genes↓, median survival of mice↑,xenografts tumor growth↓, myogenic differentiation↑, tumor regression Perk expression↓	[87]

Notes

a:Dbt-MYCN/indP3F cells: immortalized human myoblasts containing constitutive MYCN and inducible PAX3–FOXO1; ↑:Up-regulation: ↓:Down-regulation

In 2015, Lagutina. et al. first constructed a human ARSM-like chromosomal translocation t (1, 3) cell model using CRISPR/CAS9 technology in mouse myoblasts [82]. Scientists also used CRISPR/CAS9 to construct *DMD*-KO [83, 84] and *Dysf*-KO TE671 [85] cells with WT-TE671 human ERSM cells to study the pathophysiology of muscle diseases. For cancer gene screening, scientists applied iExCN analysis tools combined with CRISPR/Cas9 technology to successfully screen and verify dozens of human RMS cancer-related genes such as oncogenes *CDCA2, HAS2, SNAI2, WAR, EZH2, SCARA3, ARL4A, CDK6, ETV1, RAD54B, RIPK2,* and *ZFHX4*, and tumor suppressor genes *PTEN, ZRSR2,* and *TJP2*. Many of these genes are related to RSM growth and differentiation [79]. The results of this study provide new ideas for research and gene targeted therapy of RSM.

CRISPR/CAS9 technology was also used to study the function of RSM oncogenes. *HDAC3* knockout in 381 T ERMS cells significantly inhibited cell proliferation in vitro and tumor growth in vivo, and resulted in extensive tumor differentiation in xenograft mice, suggesting the potential value of CRISPR/CAS9 technology in RSM differentiation-induction therapy [86]. To investigate the role of the PAX3-FOXO1 fusion gene in the tumorigenesis and progression of RSM tumors, immortalized human myoblasts with doxycycline-inducible *PAX3-FOXO1* and constitutive *MYCN* expression constructs were injected into nude mice, and rapid RSM tumors formation was observed, In contrast, myoblasts expressing only *PAX3-FOXO1* formed tumors after a longer latency period. Although doxycycline withdrawal resulted in tumor regression, most tumors relapsed without induction of doxycycline. When *PAX3-FOXO1* in primary tumor-derived cell lines was knocked out by CRISPR/CAS9, cell oncogenicity disappeared and these cell lines were differentiated following doxycycline withdrawal. However, recurrent tumor-derived cell lines with *PAX3-FOXO1* knockout did not differentiate under these conditions. These findings indicate that *PAX3-FOXO1* interacts with *MYCN* to promote the occurrence of RSM by inhibiting myogenic differentiation and cell death, and recurrent tumours develop in a *PAX3-FOXO1*-independent manner [80].. In addition, the *RAS* gene may be another promising target for RSM therapy, as demonstrated by significantly reduced xenograft tumor growth and increased tumor cell death, myogenic differentiation and survival in tumor-bearing mice after the elimination of *RAS* by CRISPR/CAS9. It is worth noting that in this study, a novel CRISPR/CAS9 MYXV vector delivery system can effectively achieve targeted knockout of the oncogene *RAS* in tumors of ERMS transplanted mice [87]. This opens up the possibility of providing a new method to solve the problem of in-vivo delivery of CRISPR/CAS9.

In general, CRISPR/CAS9 technology has demonstrated great application potential in RSM research, especially in tumor differentiation-induction therapy, and more research is needed in the future to support its conversion to clinical treatment of RSM.

### Application challenges of CRISPR/CAS9

Given its high genome editing efficiency, CRISPR/CAS9 is not only used to research musculoskeletal malignancies, but also widely used in other human cancers and diseases. The application of CRISPR/CAS9 techniques to screen for pathogenic genes and therapeutic drugs, determine tumor resistance mechanisms, establish tumor models and study the pathogenesis of tumors has undoubtedly brought renewed hope to cancer patients. However it is undeniable that this new technology has just started in human disease research, and there are still many problems and challenges, which are mainly reflected in off-target effects and the efficiency and safety of in vivo delivery systems.

The main reason for the off-target effect is that Cas9-sgRNA has sequence mismatch tolerance to target DNA. The same or similar DNA sequences exist in human genome; when Cas9-sgRNA incorrectly recognizes and binds non-target DNA sequences, redundant cutting can be produced, resulting in chromosome rearrangement or off-target mutation [16]. To optimize the precision and specificity of CRISPR/Cas9 genetic modification, scientists have made many efforts in recent years to continuously develop new methods. Generally, there are two methods to reduce the off-target effect: artificially modifying the Cas9 protein or designing new gRNAs [88]. For example, scientists have described a high-fidelity variant of commonly used SpCas9, SpCas9-HF1, that reduces non-specific DNA contact, which maintains similar targeting activity while significantly reducing off-target genome editing compared to the widely used wild-type SpCas9 [89]. Similarly, scientists developed Sniper-Cas9 using *E. coli*, which also effectively increased the specificity of the CRISPR/Cas9 system [90]. There are many other studies aimed at reducing off-target effects, and we believe this challenge of CRISPR/Cas9 will be effectively solved in future research.

Another challenge for the transfer of CRISPR/Cas9 technology to clinical applications is the in vivo delivery of gene-edited elements. There are currently two main delivery routes: viral vector delivery and physical delivery. The former includes retroviruses, lentiviruses and adenoviruses, with high in vivo delivery efficiency; however, there are concerns about their potential safety and immunogenic risks. The latter includes electroporation and hydrodynamics with relatively high safety but elevated delivery efficiency is needed [16]. It is worth noting that in recent years, scientists have developed a PEG-PEI-cholesterol (PPC) lipopolymer delivery system to successfully achieve targeted delivery of gene editing elements into xenograft tumor in vivo and achieved approximately 50% gene knockouts in osteosarcoma cells, mainly by delivering the LC09 lipopolymer encapsulating the plasmid encoding *VEGFA* gRNA and Cas9 to osteosarcoma in situ and lung metastatic sites. The *VEGF* gene was effectively edited; both the malignant progression of osteosarcoma and lung metastasis were significantly suppressed without toxicity [59]. This success greatly encouraged the application of CRISPR/Cas9 in malignant musculoskeletal tumors. However, the existing research is insufficient to support the clinical transformation of CRISPR/Cas9 technology. There is still a need to further improve the efficiency of targeted delivery of non-viral methods, or to solve the immunogenicity and safety issues of viral vector delivery systems in the future.

## Discussion and outlook

Musculoskeletal malignancies are serious threats to the lives and health of adolescents and children. In addition to osteosarcoma, ES and rhabdomyosarcoma described in this review, it also includes chondrosarcoma and synovial sarcoma, etc. Other risk factors such as high birth weight and adolescent hormones may be related to the occurrence of musculoskeletal sarcoma, but adverse genetic variations must be the most fundamental cause of these diseases. Therefore, it is particularly important to explore the pathogenic genes related to tumorigenesis and development. If scientists can effectively use CRISPR/Cas9 technology to accurately edit these cancer-related genes, there is new hope for the treatment of malignant musculoskeletal tumors. Compared with traditional gene editing technology, CRISPR/Cas9 technology is easy to use, more efficient and cheaper. This technology has attracted the attention of scientists and promoted its rapid development in the research of various human diseases, and some breakthroughs have been made in the clinical application of some tumors, such as ALL. In addition, some progress has been made in musculoskeletal sarcoma, including oncogene knockout, anticancer drug screening, drug resistance mechanism research, oncogene or tumor suppressor gene screening, and disease modelling. However, there are few reports of the use of CRISPR/Cas9 for musculoskeletal sarcoma gene detection. This may be a research direction that requires further attention. Most importantly, scientists should focus on further development of more efficient and safer CRISPR/Cas9 technology and promote its transformation into clinical use for musculoskeletal malignancy patients as soon as possible. It is clear that CRISPR/Cas9 technology can provide a promising future for malignant musculoskeletal sarcoma patients and potentially revolutionize the clinical treatment of musculoskeletal malignancies.

## Conclusion

Current evidence showed that CRISPR/CAS9 technology was widely used in the field of basic research of musculoskeletal tumors, including screening and knockout of oncogenes or drug-resistant genes, identification of tumor suppressor genes and construction of cell or animal tumor models. And its technical advantages should be utilized to achieve satisfactory clinical treatment outcomes in the future.

## Data Availability

Data sharing is not applicable to this article as no datasets were generated or analysed during the current study.
